# A modular steroid-inducible gene expression system for use in rice

**DOI:** 10.1186/s12870-019-2038-x

**Published:** 2019-10-15

**Authors:** Daniela Vlad, Basel Abu-Jamous, Peng Wang, Jane A. Langdale

**Affiliations:** 1 Department of Plant Sciences, University of Oxford, South Parks Rd, Oxford, OX1 3RB UK; 2 Present address: Sensyne Health, Schrödinger Building, Heatley Road, Oxford Science Park, Oxford, OX4 4GE UK; 3 Present Address: Institute of Plant Physiology and Ecology, Shanghai Institutes for Biological Sciences, Chinese Academy of Sciences, Shanghai, 200032 China

**Keywords:** Golden gate, Dexamethasone, *Oryza sativa*, *pOp6*, LhGR

## Abstract

**Background:**

Chemically inducible systems that provide both spatial and temporal control of gene expression are essential tools, with many applications in plant biology, yet they have not been extensively tested in monocotyledonous species.

**Results:**

Using Golden Gate modular cloning, we have created a monocot-optimized dexamethasone (DEX)-inducible *pOp6*/LhGR system and tested its efficacy in rice using the reporter enzyme β-glucuronidase (GUS). The system is tightly regulated and highly sensitive to DEX application, with 6 h of induction sufficient to induce high levels of GUS activity in transgenic callus. In seedlings, GUS activity was detectable in the root after in vitro application of just 0.01 μM DEX. However, transgenic plants manifested severe developmental perturbations when grown on higher concentrations of DEX. The direct cause of these growth defects is not known, but the rice genome contains sequences with high similarity to the LhGR target sequence *lacO,* suggesting non-specific activation of endogenous genes by DEX induction. These off-target effects can be minimized by quenching with isopropyl β-D-1-thiogalactopyranoside (IPTG).

**Conclusions:**

Our results demonstrate that the system is suitable for general use in rice, when the method of DEX application and relevant controls are tailored appropriately for each specific application.

## Background

The study of gene function in plants relies on reverse genetic tools that facilitate overexpression of transgenes or suppression of endogenous gene expression. For some genes, neither approach is feasible due to the development of non-viable phenotypes. To enable functional analysis in such cases, several chemically inducible systems have been established to control transgene expression (for reviews see [1–4]). Chemically inducible systems generally consist of chimeric transcription factors and cognate promoters that are derived from genetic elements of heterologous organisms (to avoid interference with expression of endogenous genes) [2]. The dexamethasone (DEX)-inducible *pOp6*/LhGR gene expression system [5, 6] is based on a modified *Escherichia coli* lac-repressor system [7]. The *pOp6* promoter contains a concatemerized binding site comprised of six direct repeats of the 18 base pair *lac* operator (*lacO*) sequence [5]. This site is bound by the chimeric transcription factor LhGR, which is a fusion between the high affinity DNA-binding domain of the mutant *lac* repressor, *lacI*^His17^, the Gal4 transcription-activation-domain-II and the DEX-binding domain of the rat glucocorticoid receptor (GR) fused at the N-terminus. Addition of DEX to transgenic plants containing both LhGR and *pOp6* leads to relocation of LhGR from the cytoplasm to the nucleus, and consequent transcriptional activation of transgene sequences linked to *pOp6*.

Chemically inducible gene expression systems have been predominantly tested in dicotyledonous species [1–4]. However, two steroid-inducible systems have been tested in rice - the DEX-inducible *Gal4/* GVG [8] and the estrogen-inducible *pLex/* XVE [9–12]. Activation of *Gal4/* GVG in rice led to tightly controlled transgene expression but severely perturbed plant growth after exposure to moderate concentrations of inducer. Similar detrimental effects were reported in other species [13–15]. The *pLex/* XVE system was used more successfully to induce expression in rice callus, roots and leaves but different application methods were required in each case due to inefficient estradiol uptake via the roots. The more recently developed *pOp6*/LhGR system has not yet been tested in monocots but because the *pOp* promoter is not activated by endogenous factors, at least not in maize [2, 16], it may provide a suitable alternative to *pLex/* XVE.

To create a versatile inducible system for transgene expression in monocots, we developed a version of *pOp6*/LhGR using the Golden Gate modular cloning system [17]. The Golden Gate system is designed to provide a rapid, modular and scar-less assembly of large constructs, offering a flexible choice of promoters and selection modules. Tests of the system in stable transgenic rice lines revealed tight temporal control over transgene expression and the reliability of a range of approaches for induction.

## Results

### Construct design

**Fig. 1:**
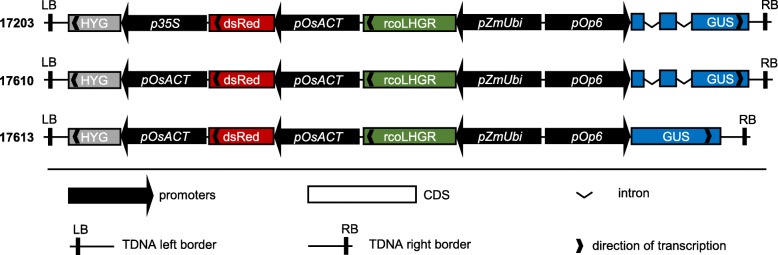
Schematic illustration of the constructs generated in this study. Plasmids 17203, 17610 and 17613 were created using the Golden Gate modular cloning system (see Additional files 2, 3, 4 for complete sequence of each). HYG: hygromycin phosphotransferase; *p35S*: *CaMV 35S* promoter; *pOsAct*: rice actin promoter; *pZmUbi*: maize ubiquitin promoter; GUS: *uidA* gene encoding β-glucuronidase. The inducible system consists of the activator, i.e. a rice codon optimized (rco) version of LhGR plus its cognate *pOp6* promoter. In each construct, all four promoter:coding sequence modules contain the *nos* terminator (not represented)

To create inducible constructs, the *pOp6* inducible promoter and the sequence encoding the corresponding chimeric transcriptional activator (*LhGR*), were synthesized as Golden Gate compatible level 0 modules, ‘PU’ and ‘SC’ [17] respectively. *LhGR* was codon optimized for use in rice (rco*LhGR*) and further ‘domesticated’ to remove all recognition sites for type II restriction enzymes used in Golden Gate cloning: BsaI, BpiI, Esp3I and DraIII (Additional file 1). Promoters, open reading frames and terminator sequences synthesized as level 0 modules were combined into standard Golden Gate level 1 vectors to generate individual transcriptional units for antibiotic selection, transcriptional activation and reporter gene expression. Three level 2 versions of the inducible Golden Gate construct were generated, differing in the choice of selection module and in the reporter gene sequence used. The three versions were designed to test the potential of the full length *CaMV 35S* promoter (*p35S*) and/or of any introns present in the reporter gene sequence, to activate expression from the *pOp6* promoter. In all cases, four level 1 modules were combined into level 2 constructs for rice transformation. All three level 2 constructs contained a dsRed reporter gene driven by the constitutive rice actin promoter (*pOsACT*) in position 2 (to allow identification of transgenic seed on the basis of fluorescence), and the rco*LhGR* gene expressed under the control of a maize ubiquitin promoter that contains an intron (*pZmUbi*) [18] in position 3. In position 1, the first construct (17203) contained *p35S* driving expression of the hygromycin-resistance selectable marker gene *hygromycin phosphotransferase* (*HYG*), whereas constructs 17610 and 17613 had *pOsACT* driving expression of *HYG*. In position 4, the *E. coli uidA* gene encoding the enzyme β-glucuronidase (GUS) was used as a reporter for *pOp6* promoter activity. In constructs 17203 and 17610, the *uidA* sequence contained two introns from the *GFA1* (At1g06220) gene in Arabidopsis, whereas the sequence in construct 17613 was intron-less (Fig. 1; Additional files 2, 3, 4).

### The *pOp6*/rcoLhGR system is functional in rice

To test the *pOp6*/rcoLhGR system in a monocot species, all constructs were transformed into rice (*Oryza sativa spp japonica* cv Kitaake) to obtain stable transgenic lines. Positive T0 transformants were first validated by histochemical GUS detection after DEX induction of detached leaf fragments (data not shown), and then transgene copy number was assessed by DNA gel blot analysis (Additional file 5). Lines with one or two copies of the transgene plus line 17613_11, for which insertion copy was not determined in T0 plants, were propagated into the T1 generation. At least four independent T1 lines were obtained for each of the three constructs, with transgene copy number ranging from one to three (Additional file 6).

**Fig. 2:**
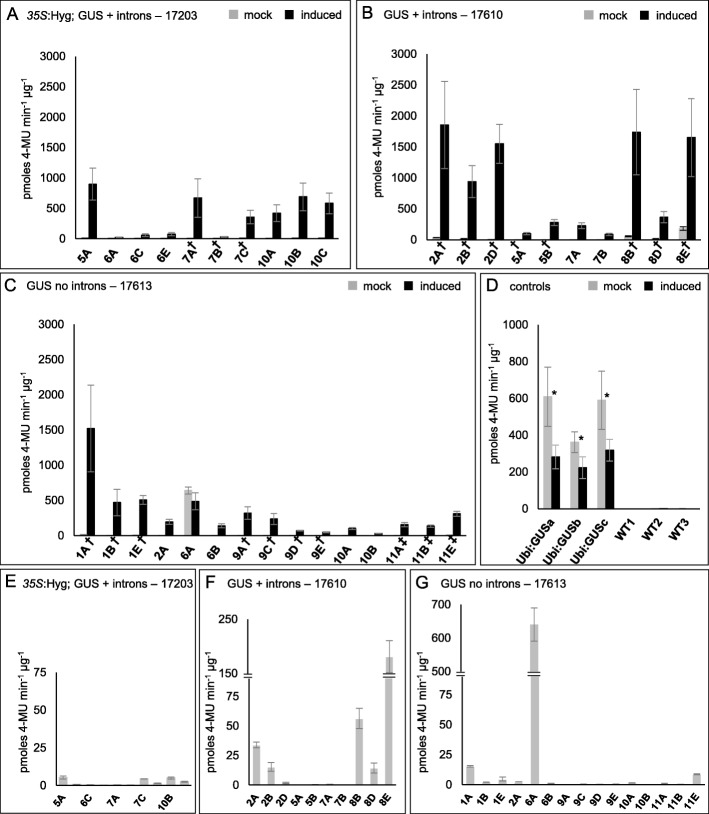
GUS activity is induced by DEX in 14 independent T1 *pOp6*/rcoLhGR lines. GUS enzymatic activity measured in total leaf protein extracts from individuals segregating in T1 lines transformed with construct 17203 (**a**, **e**), 17610 (B, F) or 17613 (**c**, **g**). Individuals from a *pZmUbi*:*GUS* line were used as positive controls, and wild-type Kitaake rice plants (WT) were used as negative control (**d**). The assay used 4-methylumbelliferyl β-D-glucuronide (4-MUG) as a substrate to detect activity in the absence (grey bars) or presence (black bars) of 10 μM DEX. Bars represent the mean value from 3 technical replicates per individual ±SD. *P < 0.05 in a paired t-test. Note the maximum value on the y-axis is 3000 in (**a**-**c**), 1000 in (**d**) 75 in (**e**), and the axis is discontinuous in (**f**, **g**). Raw data are presented in Additional file 7. Nomenclature used: number indicates independent transgenic event and letters indicate segregating T1 individuals derived from each event. As such, at least four independent events were tested for each construct: lines 5, 6, 7 and 10 for 17203, lines 2, 5,7 and 8 for 17610 and lines 1, 2,6, 9, 10 and 11 for 17613. † indicates lines segregating for two linked T-DNA insertions, ‡ indicates lines segregating for three T-DNA insertions and the remaining lines have a single insertion

The efficacy of the *pOp6*/rcoLhGR system in rice was first assessed by measuring GUS activity using an extractable enzymatic assay. Leaves were detached from individuals of at least four independent T1 lines per construct, treated with DEX for 24 h and then assayed for GUS activity using the substrate 4-methylumbelliferyl ß-D-glucuronide (MUG) (Additional file 7). A line that constitutively expresses a synthetic GUS variant from *Staphylococcus* (GUSPlus) under the control of the maize ubiquitin promoter (*pZmUbi*:*GUS*) was used as a positive control for the fluorometric MUG assay. Figure 2 shows that DEX induction often resulted in higher levels of GUS activity in T1 individuals transformed with the inducible constructs than in non-induced individuals of the constitutive *pZmUbi*:*GUS* line (compare Fig. 2a-c ‘induced ‘with 2D ‘mock’). Given that GUSPlus is reported to be ten-fold more active than the enzyme encoded by *E. coli uidA* that was used in the inducible constructs [19, 20], this observation suggests that activation of the *pOp6* promoter by the rcoLHGR is very effective. Curiously, GUS activity in the *pZmUbi*:*GUS* line was suppressed in the presence of DEX (Fig. 2d) suggesting a possible inhibitory interaction between the steroid and the synthetic GUS enzyme in the fluorometric assay. Importantly, in all but one of the 14 inducible lines tested, GUS activity was significantly higher after DEX application, with very little activity detected in the absence of the inducer (Fig. 2a-c). Even in the exceptional line (17613–6), high levels of background activity were only observed in one of the two individuals examined (17613-6A, Fig. 2c). There was no correlation between transgene copy number and levels of GUS activity before or after induction. For example, line 17203_10 was segregating for a single T-DNA insertion and line 17203_7 for two (linked) insertions (Additional file 5), yet levels of GUS activity after DEX induction were similar in both lines (Fig. 2a). Variation in activity between individuals within T1 families may be explained by variable zygosity resulting from segregation of the transgene. Together these results indicate that the *pOp6*/rcoLhGR system is functional, sensitive, and tightly regulated in rice.

To determine whether potential enhancer sequences in the *p35S* promoter or in introns in the GUS reporter influence the fidelity of the *pOp6*/rcoLhGR system, comparisons were first made between GUS activity detected in lines transformed with construct 17203 (*p35S*:Hyg; GUS + introns) (Fig. 2a) and those with 17610 (no *p35S*; GUS + introns) (Fig. 2b). Although levels of GUS activity after DEX induction appeared lower in the presence (Fig. 2a) than the absence (Fig. 2b) of *p35S*, the difference was not statistically significant by a Wilcoxon’s rank test *P* > 0.05 (Additional file 7). As such, the presence of the *p35S* promoter (and of any enhancers within it) did not influence reporter gene expression. This conclusion was validated by the observation that very little ‘leaky’ reporter gene expression was observed in the absence of inducer (Fig. 2e). It remains possible, however, that construct designs that bring the *pOp6* inducible promoter closer to *p35S* might result in transgene activation in the absence of DEX, for example as in Yoo et al. [21]. Similar comparisons between GUS activity levels in lines transformed with construct 17610 (no *p35S*; GUS + introns) (Fig. 2b, f) and those with 17613 (no *p35S*; GUS no introns) (Fig. 2c, g) revealed that the presence of introns in the reporter gene had an enhancing effect on activity levels both in the absence and presence of inducer. In the presence of inducer, this difference was statistically significant in a Wilcoxon’s rank test at *P* < 0.05 (Additional file 7). These results suggest that the intron sequences enhanced transcription from the *pOp6* promoter when it was bound by LhGR. Collectively, these data demonstrate that although rcoLhGR reliably responds to steroid induction, transcription from the activated *pOp6* promoter may be influenced by regulatory sequences elsewhere in the construct, and particularly by introns in the reporter gene of interest.

### Dexamethasone in the culture medium inhibits growth of transgenic rice seedlings

**Fig. 3:**
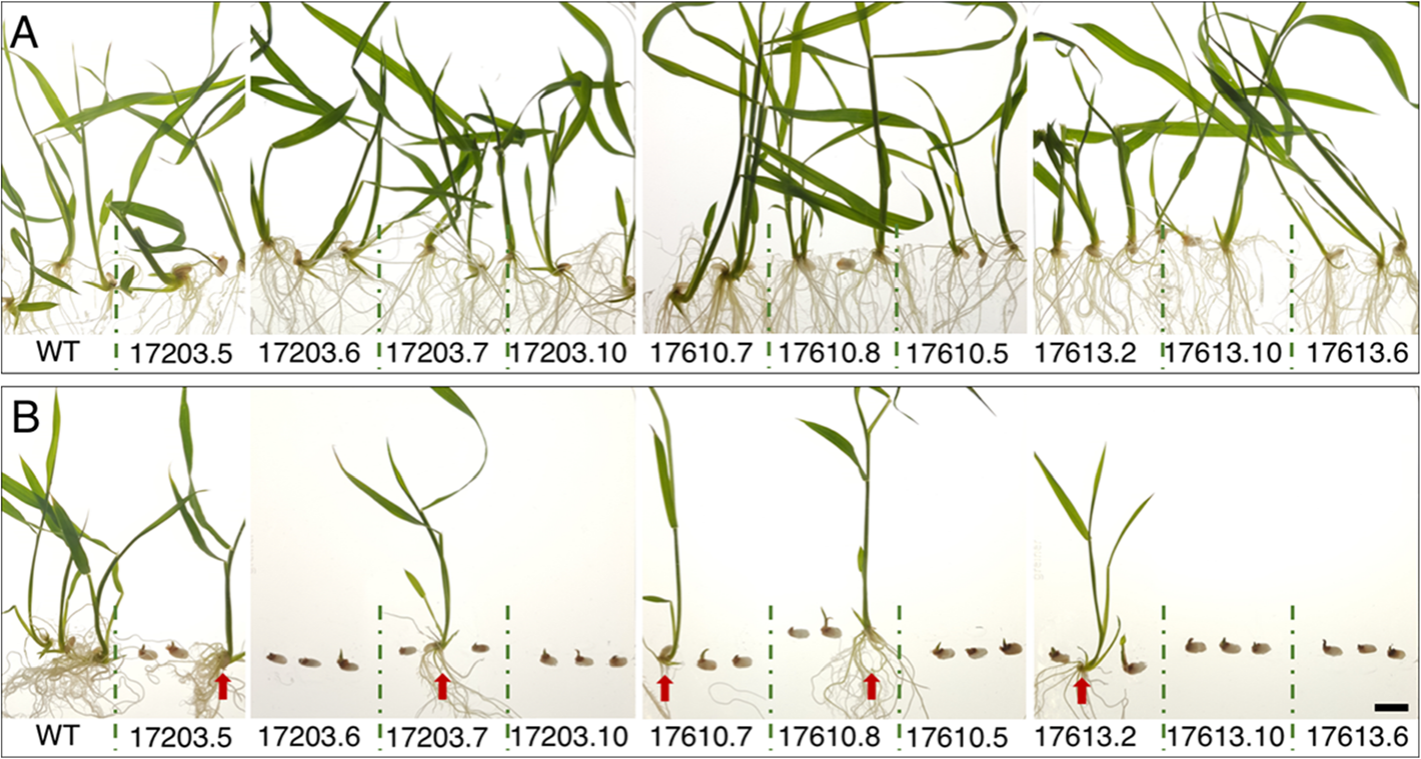
Seedling growth on medium containing DEX is severely compromised. Seeds from 10 independent inducible lines were germinated and grown on ½ MS medium without (**a**) and with (**b**) 10 μM DEX for 12 days. Some lines segregated seedlings that were not inhibited by DEX (red arrows). Scale bar = 1 cm

Because induction of reporter gene expression may be required at early developmental stages in some experiments, we tested whether *pOp6*/rcoLhGR transgenic lines could be reliably germinated on medium containing DEX. For at least three independent T1 lines per construct, three seeds per line were germinated and grown on plates containing ½ MS medium, either with or without 10 μM DEX. All 30 transgenic plants grown on plates without DEX were indistinguishable from wild-type controls (Fig. 3a). However, of the 30 seed germinated in the presence of 10 μM DEX, only 5 grew normally (Fig. 3b), and these were likely to be null segregants that did not contain the *pOp6*/rcoLhGR transgene. The remaining seedlings were severely stunted, with shoots only a few millimetres tall after 12 days. Together these results suggested that the *pOp6*/rcoLhGR transgene severely inhibited growth in the presence of 10 μM DEX.

Given that plant growth was only compromised by DEX in lines where the transgene was present, we hypothesized that LhGR was activating the expression of endogenous rice genes that inhibit seedling growth. The *lacI* binding domain of LhGR was designed to specifically bind an 18-bp *lacO* sequence in the *pOp6* inducible promoter [5]. However, *lacI* also has the potential to bind to *lacO*-related sequences found in the plant genome, and has been shown to do so even with 5/18 mismatches in the target site [22]. To determine the frequency of potential off-target sites in the rice genome, a bioinformatic analysis was carried out to identify *lacO* sequences with 0–5 mismatches. A total of 2556 potential binding sites were identified, of which 125 were positioned within 1 kb of an open reading frame (Table 1). DEX-induced binding of LhGR to any of these 125 putative promoter sequences could cause growth defects, depending on the function of the downstream coding sequence.

**Table 1:** Occurrence of *lacO*-related sequences in the rice genome

*lacI* wild type binding sites	number of hits in the rice genome (putative promoters)			
	exact matches	3 mismatches	4 mismatches	5 mismatches
*lacO1* AATTGTGAGCGGATAACAATT	1	4	111 (4)	1144 (43)
*lacO2* AAATGTGAGCGAGTAACAACC	0	6 (1)	88 (3)	709 (35)
*lacO3* GGCAGTGAGCGCAACGCAATT	1	3	53 (3)	436 (38)

The three *E. coli lacO* sequences were used to query the rice genome, with criteria set to return all hits with a maximum five mismatches (all of which are predicted to bind *lacI* – based on binding studies in tobacco ([22])

**Fig. 4:**
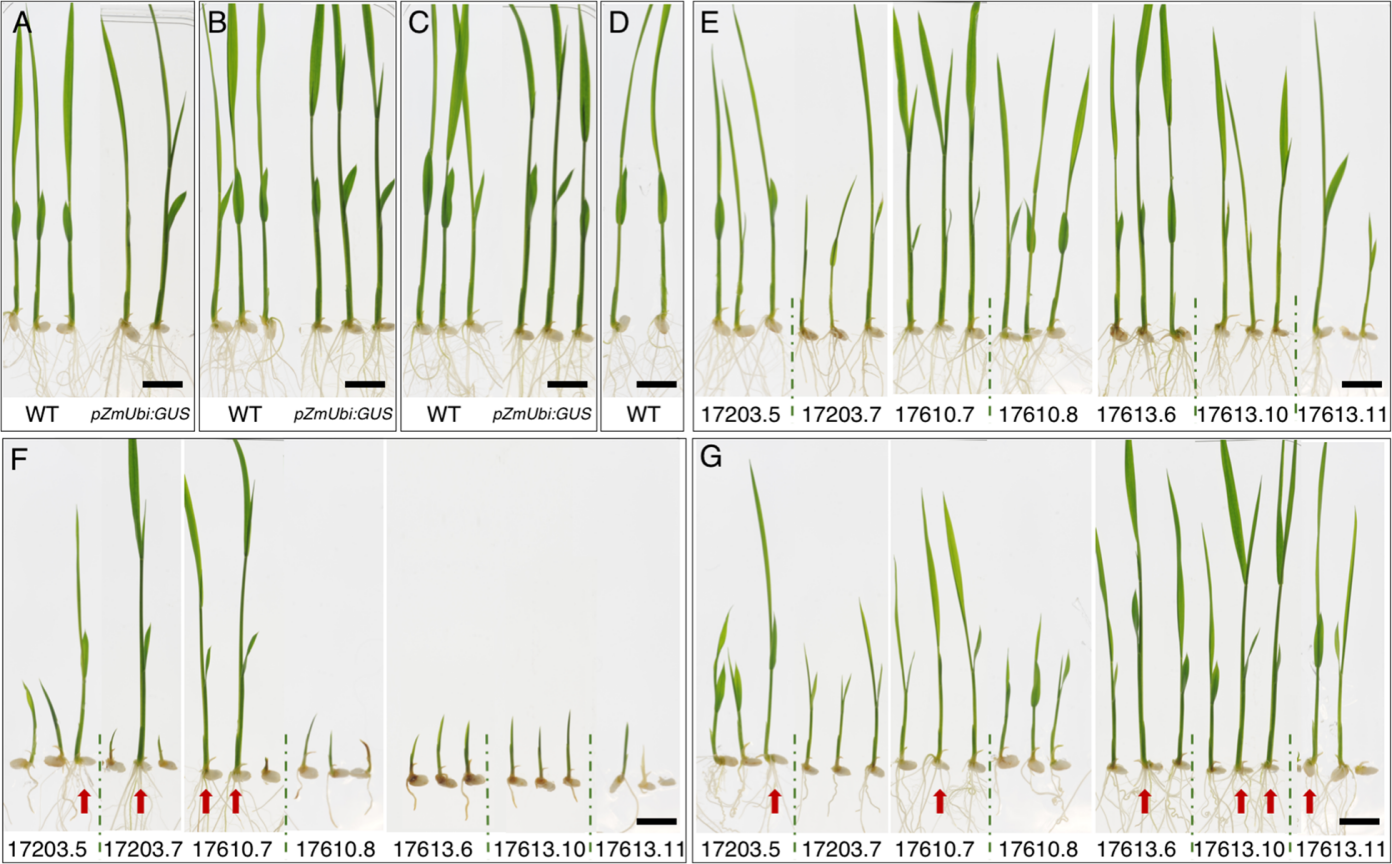
DEX-induced growth defects in transgenic lines can be ameliorated by addition of IPTG. a-**d**) *pZmUbi:GUS* and wild-type controls. **e**-**g**) T1 seedlings from 7 independent transgenic lines (at least two lines each for the inducible constructs 17203, 17610 and 17613). All seeds were germinated on ½ MS plates for 3 days and then transferred to plates containing either: 10 mM IPTG (**a**, **e**): 10 μM DEX (**b**, **f**): 10 μM DEX plus 10 mM IPTG (**c**, **g**); or ½ MS (**d**) and grown for 5 days. Some lines segregated non-transgenic siblings which were not affected by DEX-mediated LhGR activation (marked by red arrows and confirmed by GUS staining in Fig. 6). Scale bar = 1 cm

Given that the *lacI* binding site of LhGR can be bound by isopropyl β-D-1-thiogalactopyranoside (IPTG) [5], we tested whether off-target activation could be ameliorated by addition of 10 mM IPTG to the DEX-containing growth medium. Using at least two independent lines for each construct, 3 seeds per T1 line were pre-germinated for 3 days on ½ MS before transferring to either 10 μM DEX, 10 μM DEX plus 10 mM IPTG, or 10 mM IPTG alone. Both wild-type and *pZmUbi:GUS* lines were grown alongside as controls. Figure 4 shows that seedling growth in both wild-type and *pZmUbi:GUS* lines was equivalent in all three conditions (Fig. 4a-c), and that growth of wild-type plants was not affected by 10 μM DEX (compare Fig. 4b and d). Growth of inducible lines on 10 mM IPTG alone was also essentially normal, although there some heterogeneity between seedlings in lines 17203.7 and 17613.11 (Fig. 4e). In contrast, seedlings of all 7 inducible lines showed severe growth defects on 10 μM DEX (Fig. 4f). Note that most of the T1 lines were segregating non-transgenic siblings (17203.5; 17203.7; 17610.8 – red arrows; identified by lack of GUS staining – see Fig. 6e) that were not affected by DEX. Notably, the degree of growth perturbation was similar in all inducible lines, regardless of the construct. This is consistent with the fact that all constructs contain LhGR driven by the same promoter (*pZmUBI*), and that the growth assay is recording activation of off-target sites as opposed to activation of the *pOp6:GUS* reporter gene. Importantly, growth defects were ameliorated when seedlings of inducible lines were grown on DEX plus IPTG (Fig. 4g), particularly in lines 17613.6, 17613.10 and 17613.11 which contain 1, 2 and 3 transgene copies respectively. In general, shoot size was at least doubled in the presence of IPTG. Collectively, these results demonstrate that growth perturbations on DEX are a direct consequence of LhGR activity, and that off-target effects can be quenched by IPTG.

### The *pOp6* promoter can be induced by levels of dexamethasone that do not inhibit growth

**Fig. 5:**
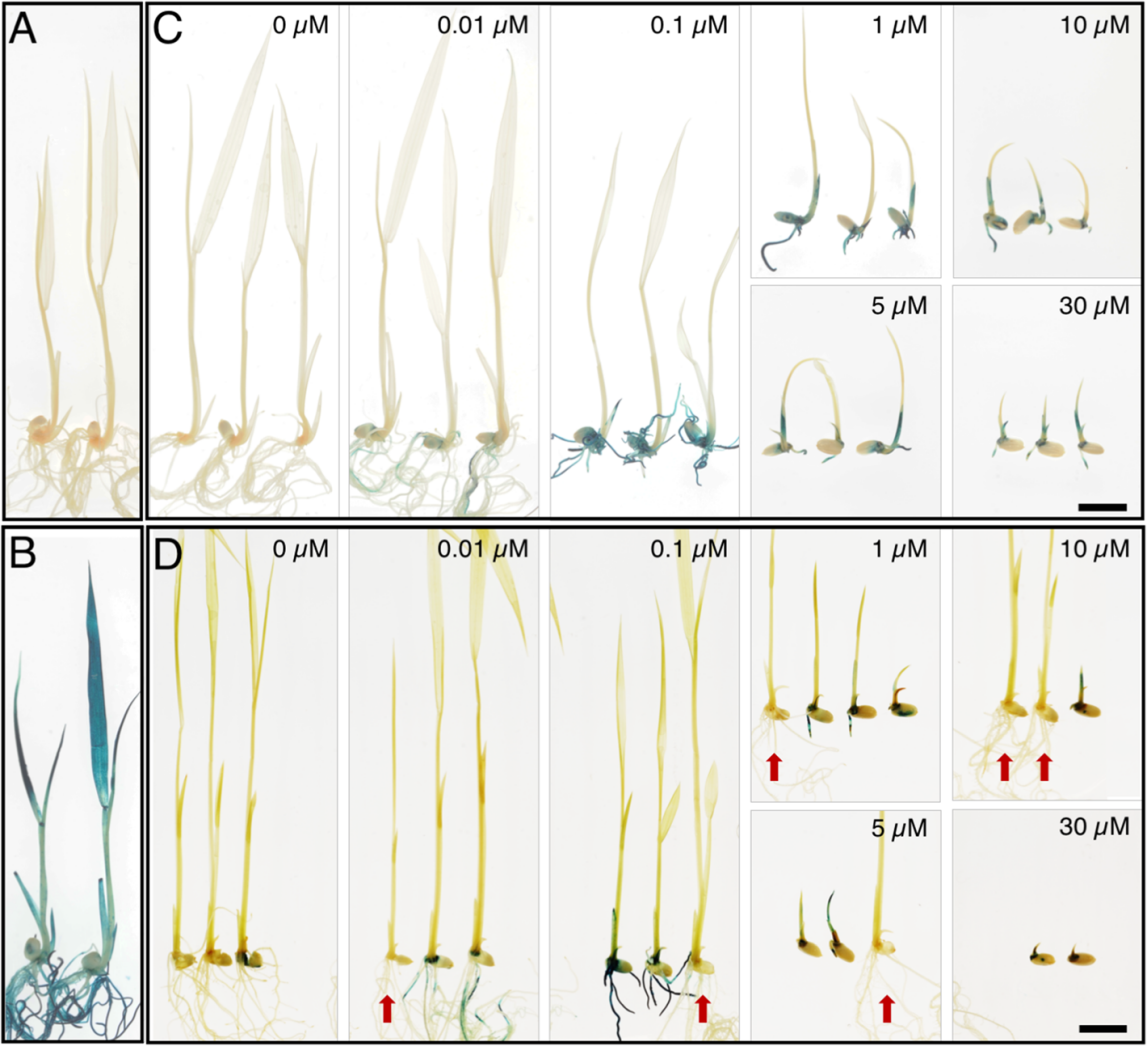
*pOp6*-driven reporter gene expression can be induced by DEX concentrations that do not inhibit plant growth. **a**-**b**) Wild-type (**a**) and *pZmUbi:GUS* (**b**) controls germinated and grown on ½ MS. **c**, **d**) T1 seedlings from inducible lines 17613.11 (**c**) and 17203.6 (**d**) germinated on ½ MS for 3 days before transferring to plates with 0, 0.01, 0.1, 1, 5, 10 and 30 μM DEX for 5 days before staining for GUS activity. Some lines segregated non-transgenic siblings which were not affected by DEX-mediated LhGR activation (marked by red arrows). Scale bar = 1 cm

**Fig. 6:**
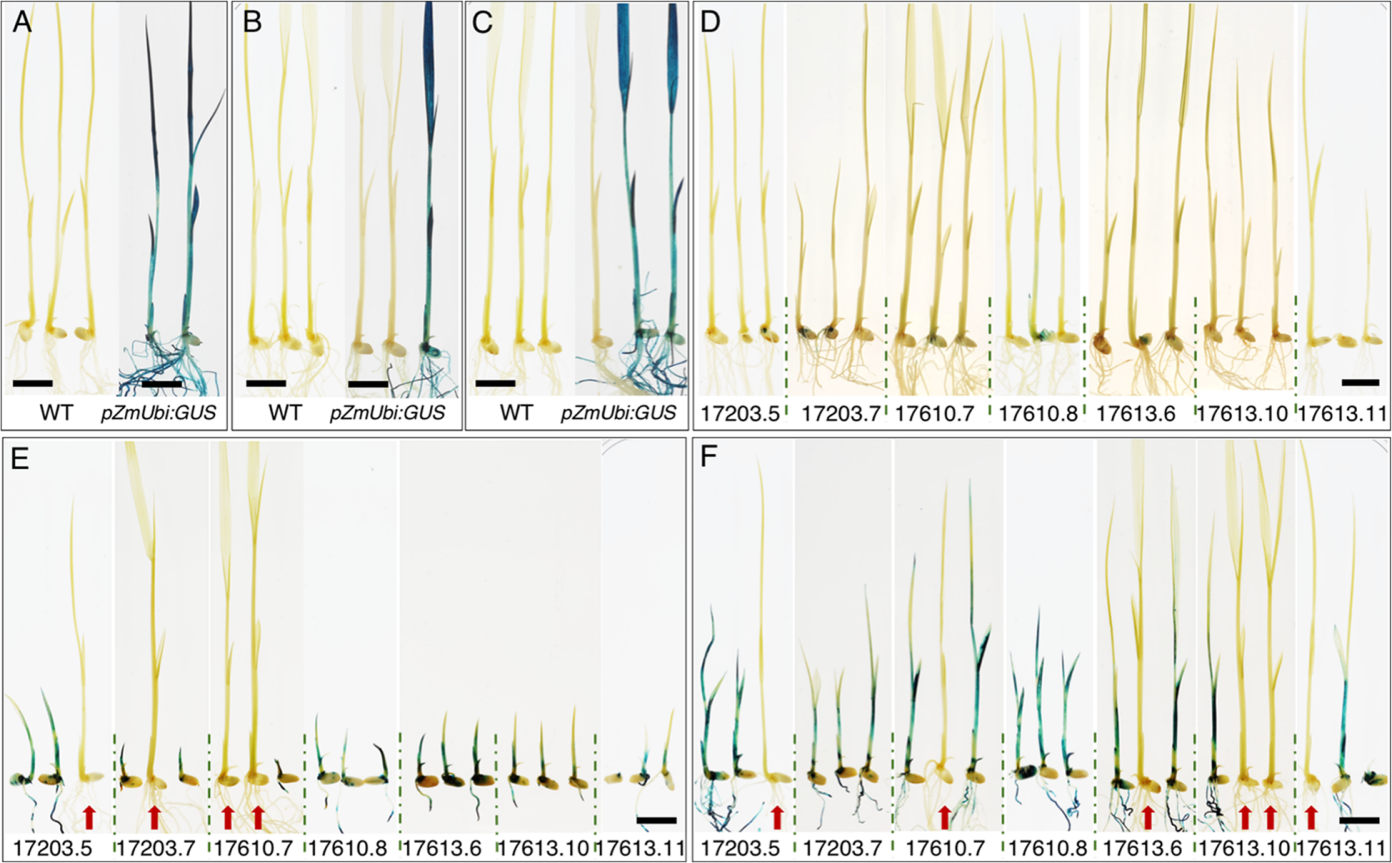
Addition of IPTG to the growth medium does not inhibit activation of *pOp6*-driven reporter gene expression. Seedlings shown in Fig. 4 stained for GUS activity. **a**-**c**) *pZmUbi:GUS* and wild-type controls. **d**-**f**) T1 seedlings from independent inducible lines. All seeds were germinated on ½ MS plates for 3 days and then transferred to plates containing either: 10 mM IPTG (**a**, **d**): 10 μM DEX (**b**, **e**) or 10 μM DEX plus 10 mM IPTG (**c**, **f**) and grown for 5 days. Some lines segregated non-transgenic siblings which were not affected by DEX-mediated LhGR activation (marked by red arrows). Scale bar = 1 cm

To determine whether *pOp6*-driven reporter gene expression can be induced by DEX concentrations that do not inhibit growth, T1 seeds derived from lines 17203.6 and 17613.11 were pre-germinated on ½ MS plates and then after 3 days transferred to the same medium containing various concentrations of DEX (0.01, 0.1, 1, 5, 10, and 30 μM). These two inducible lines represent different transgene copy numbers (1 versus 3) and different reporter gene composition (with versus without introns). Despite these differences, Fig. 5 shows consistent effects of DEX on seedling development and on the level of GUS activity that can be detected. Seedlings of wild-type (Fig. 5a), *pZmUbi:GUS* (Fig. 5b) and of both inducible lines (Fig. 5c, d) grew to an equivalent size in the absence of DEX, and only the *pZmUbi:GUS* plants stained positive for GUS, with activity detected throughout the plant (Fig. 5b). By contrast, growth was severely compromised in seedlings from the inducible lines that stained positive for GUS (i.e. the plants containing the transgene) when plants were grown on 1, 5, 10 or 30 μM DEX (Fig. 5c, d). Note that the seedlings that grew normally in these conditions did not stain for GUS and were thus null segregants. Growth of inducible lines was normal on 0.01 μM DEX and only partially compromised on 0.1 μM DEX (where roots were shorter than untreated plants), however, GUS activity was only induced in the roots (0.01 μM DEX) or in the roots plus the lower parts of the shoot (0.1 μM DEX) (Fig. 5c, d). Although this induction indicates that the *pOp6*/rcoLhGR inducible system is very sensitive, the low levels of activity induced at these concentrations may not be sufficient for some applications. We therefore tested whether the levels of IPTG used to quench growth defects caused by exposure to 10 μM DEX (Fig. 4) affected levels of inducible GUS activity. To this end, the individuals shown in Fig. 4, which had been grown in the presence of 10 μM DEX, with or without added 10 mM IPTG, were stained for GUS activity. Figure 6 shows the presence of GUS activity throughout *ZmUbi:GUS* plants (Fig. 6a-c), whereas no activity was detected in wild-type plants (regardless of growth treatment) (Fig. 6a-c) or in inducible lines grown on 10 mM IPTG (Fig. 6d). Similar levels of GUS activity were detected in inducible lines grown on 10 μM DEX both in the absence (Fig. 6e) and presence (Fig. 6f) of 10 mM IPTG. This result suggests that for any individual transgenic line, it should be possible to optimize DEX and IPTG concentrations in the culture medium, to minimize growth defects whilst still conferring sufficient levels of induced transgene activity.

**Fig. 7:**
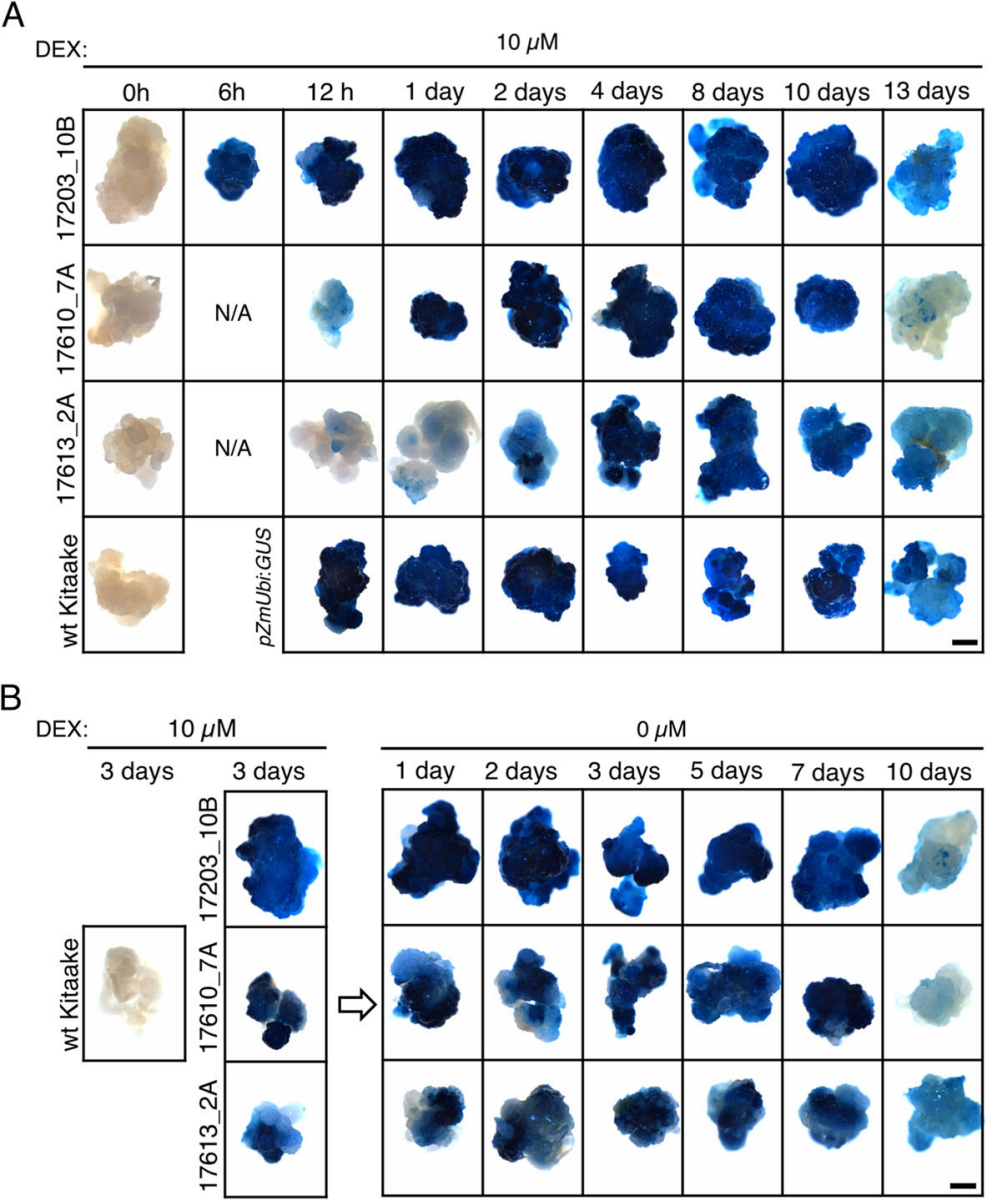
Time course of GUS activity after induction and treatment interruption in transgenic callus. **a**, **b**) Histological detection of GUS activity in transgenic and wild-type (wt) callus. Calli were grown for 13 days on solid medium containing 10 μM DEX (**a**) or induced with 10 μM DEX for 3 days and then transferred to fresh solid medium without DEX for 10 days (**b**). Scale bars = 1 mm

**Fig. 8:**
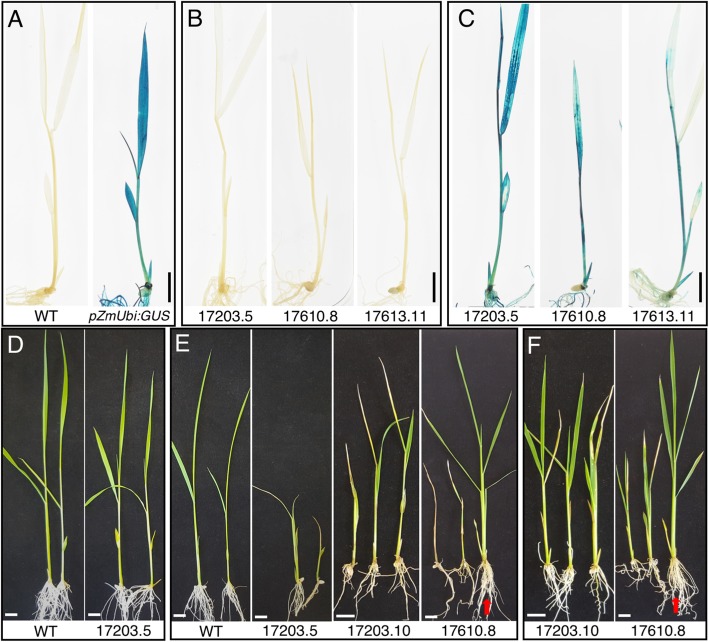
Reporter gene expression can be induced by seedling submergence. **a**-**c**) GUS activity in representative 7 day old seedlings that were germinated and grown on ½ MS plates before submergence in non-inductive (0.1% DMSO and 0.1% Tween-20) (**a**, **b**) or inductive (10 μM DEX, 0.1% Tween-20) (**c**) solutions for 19 h. D-F) Phenotype of plants transferred to hydroponic growth conditions for 5 days after submergence treatment. Prior to submergence in non-inductive (**d**) or inductive (**e**, **f**) conditions, plants had been grown in either ½ MS (**d**, **e**) or in ½ MS media containing 10 mM IPTG. Red arrows indicate segregating wild type plants. Scale bar = 1 cm

To determine whether alternative methods of DEX application can be used to induce transgene expression without inhibiting growth, a seedling submergence approach was tested. Because the *pOp6*/rcoLhGR system showed a rapid response to 10 μM DEX application in transgenic callus, a quick induction method was used. In calli, experiments with 3 independent lines (a single line for each construct), showed that GUS activity was induced within 12 h of application (Fig. 7a). Using a second line for each construct, we submerged 7 day old seedlings that had been grown on ½ MS plates into a 10 μM DEX solution for ~ 19 h, before staining for GUS activity. Figure 8 shows that no GUS activity was detected in wild-type (Fig. 8a) or mock-treated inducible (Fig. 8b) plants, whereas GUS activity was detected uniformly throughout *ZmUbi:GUS* and DEX-treated inducible plants (Fig. 8c). The line containing construct 17613 showed lower levels of activity in the leaf compared with either 17203 or 17610 lines, consistent with GUS activity levels quantified in leaf extracts (Fig. 2) and with activities in transgenic callus where maximum levels of activity were reached after 24 h in lines containing constructs with introns in the reporter gene (17203 and 17610) but required 4 days of induction when the intron-less version was present (17613) (Fig. 7a). Crucially there were no apparent morphological differences between the transgenics and wild-type controls using the submergence assay. Together with the consistently uniform GUS activity seen across tissues of the submerged plants, this observation indicates that the method is suitable for applications where phenotypes need to be assessed immediately following induction of gene expression.

In some experiments induction will be needed some days before phenotypic characterization, however, and thus to test whether the DEX submergence treatment affected subsequent growth, non-GUS-stained plants were transferred back to ½ MS medium. Plants that had been grown for 7 days on either ½ MS (Fig. 8d, e) or on ½ MS plus 10 mM IPTG (Fig. 8f) were either left in ½ MS (Fig. 8d) or submerged in 10 μM DEX (Fig. 8e, f) for 19 h. All plants were then returned to liquid ½ MS medium for 5 days. Figure 8f shows that seedlings pre-treated with IPTG recovered better after return to non-inductive conditions than those that had not been pre-treated (Fig. 8e). This suggests that DEX was retained in the plant tissues after submergence, consistent with the observation that induced callus retained high GUS activity levels for at least 5 days after removal from DEX treatment (Fig. 7b). Therefore, the submergence method is not suitable for applications where a delay is required between induction and phenotypic characterization.

## Discussion

The modular *pOp6*/LhGR glucocorticoid-inducible system reported here enables efficient and robust induction of transgene expression in rice (Figs. 2, 5, 6, 7, 8). The system is very sensitive, responding to nanomolar concentrations of DEX (Fig. 5), and is amenable to different methods of DEX application to suit different purposes. For example, detached tissues (as in Fig. 2) could be used to screen regenerating T0 lines for effective induction of transgene expression, submergence assays (as in Fig. 8) could be used to identify immediate downstream targets/effects of induced gene activity, and growth on 0.01 μM DEX-containing medium (as in Fig. 5) could be used to analyse the effect of the induced gene on root development. Controlled induction of genes during shoot development will be more challenging, however, because DEX concentrations that are required to induce the *pOp6* promoter in the shoot, inhibit growth when included in the culture medium (Figs. 3, 4 & Fig. 6). Although inclusion of IPTG in the culture medium, ameliorates the toxic effects of LhGR (Figs. 4 & 6), the relative concentrations of DEX versus IPTG will need to be calibrated for each transgenic line in order to maximize transgene expression and minimize growth defects. That is, to quench DEX-induced LhGR binding to *lacO*-like sites in the rice genome, whilst still allowing efficient binding to the *pOp6* promoter in the transgene. Crucially, very little expression was seen from the *pOp6* promoter in the absence of inducer, which will allow potentially lethal transgenes to be carried through regeneration of T0 plants, ensuring successful harvest of T1 transgenic seed.

The severe defects observed in rice *pOp6*/LhGR transgenic lines after growth on 10 μM DEX were unexpected, because similar concentrations of the steroid are not reported to have an effect in *pOp6*/LhGR lines of either tobacco or Arabidopsis [5, 6]. Indeed, the *pOp6*/LhGR system was first developed to overcome the problem that the existing DEX-inducible system, which utilized the transcriptional activator GVG with the cognate *GAL4* promoter [23], frequently led to growth defects in Arabidopsis, rice and *Lotus Japonicus* [8, 14, 15]. Although the molecular basis of these growth defects was not established, it was proposed that the GVG activator was binding to cis-regulatory elements in the plant genome that had sequence homology to *GAL4* [15]. The GVG activator comprises the DNA-binding domain of the yeast GAL4 transcription factor, the activation domain of herpes viral protein VP16 and the same GR domain as in LhGR [23]. The Gal80 inhibitor could possibly be used to titrate off-target GAL4 binding [24] in the same way that IPTG was used here as an effective antagonist for the *LacI* DNA binding domain in LhGR (Figs. 4, 6), but the *Gal80* gene would have to be co-expressed *in planta* and activity may be difficult to modulate. The *pOp6/*LhGR system reported here is thus currently the best choice for use in rice, with the caveat that phenotypic effects of induced gene expression need to be distinguished from non-specific effects of LhGR, through comparison with control lines that have similar levels of LhGR expression but no gene of interest downstream of *pOp6*.

Because of the modular Golden Gate cloning strategy that was used to generate the *pOp6*/LhGR constructs reported here, there is potential to easily modify the system for further optimization. For example, multiple genes of interest could be simultaneously induced with a single LhGR module and/or tissue-specific promoters could be added to spatially control LhGR activity. In all cases, care must be taken when introducing introns into constructs, as regulatory sequences within them may enhance gene expression both with and without induction [25]. An alternative approach would be to generate a synthetic system that is orthogonal to the rice genome and hence unable to be compromised by off-target effects of DEX application. In a synthetic system, transcriptional activators such as dTALEs [26] would be designed to bind unique promoter sequences that are not present in the rice genome, and the activator would then be fused to the GR sequence used here. Indeed, this approach could be used to design an orthogonal system for any species of choice.

## Conclusions

Our results demonstrate the uses and limitations of a monocot optimised *pOp6*/LhGR inducible gene expression system in rice. By adapting pOp6/LhGR to Golden Gate modular cloning, we created a highly versatile system conferring transgene expression in the presence of small concentrations of DEX. However, transgenic plants manifested developmental perturbations upon LhGR activation at higher DEX concentrations. We used IPTG to demonstrate that growth perturbations were a direct effect of LhGR activation and that it is possible to optimize IPTG and DEX concentration to minimize growth defects and maximize levels of induced transgene activity.

Although not fully orthogonal, the inducible gene expression system described here is suitable for general use in rice, when the method of DEX application and relevant controls are tailored appropriately for each specific application.

## Methods

### Generation of recombinant constructs

The *pOp6* inducible promoter and the corresponding chimeric transcriptional activator LhGR-N (referred to as LhGR throughout the manuscript), as reported by Samalova et al. [6], were adapted for Golden Gate (as below) and synthesized as level 0 modules, PU and SC, respectively. The *LhGR* sequence was codon optimized for use in rice (rco) and further ‘domesticated’ to remove all recognition sites for type II restriction enzymes used in Golden Gate cloning: BsaI, BpiI, Esp3I and DraIII.

Cloning was carried out using standard Golden Gate parts (Additional file 8) and the one-step one-pot protocol [27]. Golden Gate modules EC47761, EC75111, EC41421, EC47822, EC49283, EC47811 and binary vector pAGM4723 were a gift from Sylvestre Marillonnet and Nicola Patron (‘EC’ modules are referred to as ‘pICH’ in [17, 28]); modules EC15069, EC15030 containing p35S, EC15216, EC15455 and EC15073 were a gift from Ben Miller (University of East Anglia, UK) and pICSL4723 was a gift from Mark Youles (The Sainsbury Lab Norwich, UK). All EC modules were obtained from the Oldroyd lab (Sainsbury Lab, Cambridge University, UK). All promoters used in this study with the exception of *pOp6* were characterized in [18].

The *pOp6* promoter was cloned into the level 1 backbone EC47761 (position 4, forward) upstream of either a *uidA* (*GUS*) gene containing plant introns (EC75111) or a version with no introns (*kzGUS*, a gift from Dong-Yeon Lee, Donald Danforth Center, St Louis, USA). Modules were finished by addition of a *nos* terminator (EC41421). *rcoLhGR* was cloned downstream of the maize ubiquitin promoter (*pZmUbi*, EC15455) and upstream of the *nos* terminator, into level 1 backbone EC47822 (position 3, reverse).

Level 1 modules described above were assembled into the binary vector pAGM4723 (17203) or pICSL4723 (17610 and 17613) to obtain constructs depicted in Fig. 1. Each final construct contained a *hygromycin phosphotransferase* (*HYG*) selection module (EC15069) activated by either a *CaMV 35S* (*p35S*) promoter (level 1 module EC15030) or the rice actin promoter (*pOsACT*, EC15216) in position 1; a *pOsAct*-*dsRed* module (dsRed module EC15073) included to assist with transformed callus selection in position 2 (EC47811); followed by the *rcoLhGR* module in position 3 and a *pOp6* reporter module in position 4. Vectors were closed with end linker ELB4 (EC49283).

The *pZmUbi*:*GUS* construct was generated by Gateway cloning of the *GUSPlus* cDNA into the binary destination vector pVec8-Gateway [29]. *GUSPlus* cDNA was amplified by PCR from plasmid pCambia1305.2 with Gateway® compatible primers (PW61F: 5′-GGGGACAAGTTTG TACAAAAAAGCAGGCTCATGGCTACTACTAAGCATTTGG-3′; PW61R: 5′-GGGGACCACTTTGT ACAAGAAAGCTGGGTTCACACGTGATGGTGATGG-3′). The coding sequence was subcloned into Gateway® donor vector pDONR™207 in a BP reaction, sequenced, and then cloned downstream of the *ZmUbi* promoter in the binary destination vector pVec8-Gateway [29] via an LR reaction.

### Plant material and transformation

Seeds of *Oryza sativa spp. japonica* cultivar Kitaake were obtained from the International Rice Research Institute, Los Banos, Philippines. To generate stable transgenic lines, constructs were transformed into rice using *Agrobacterium tumefaciens*. Calli were induced from mature seeds before co-cultivation with *A. tumefaciens* strain EHA105 that had been transformed with the construct of interest. Callus transformation and seedling regeneration were performed at 32 °C according to a protocol modified from Toki et al. [30], that can be downloaded at https://langdalelab.files.wordpress.com/2015/07/kitaake_transformation_2015.pdf. Regenerated T0 plantlets were verified by polymerase chain reaction (PCR) using primers to detect the presence of the selection gene HYG (forward primer: 5′- CAACCAAGCTCTGATAGAGT-3′; reverse primer: 5′- GAAGAATCTCGTGCTTTCA-3′) and/or by GUS staining, and positive transformants were transferred to commercial soil based compost (J. Arthur Bower’s John Innes Compost No.2, pH 5.5–6.0, particle size < 12 mm).

### DNA gel blot analysis

Genomic DNA was isolated from 300 to 400 mg of rice leaf tissue that had been ground in liquid nitrogen using 500 μl CTAB extraction buffer (1.5% CTAB, 1.05 M NaCl, 75 mM Tris-HCl, 15 mM EDTA pH 8.0) [31]. After incubating at 65 °C for 1 h, samples were thoroughly mixed with equal volumes of chloroform:isoamylalcohol (24:1) and centrifuged at 13000 rpm for 10 min. The aqueous layer was transferred to fresh tubes, precipitated by mixing with equal volumes of isopropanol and centrifuged for 10–15 min at 13000 rpm. Pellets were washed with 70% ethanol, air-dried and dissolved in 50 μl dd H_2_O.

For each transgenic plant, 10 μg genomic DNA was digested with the *HindIII* restriction endonuclease (New England Biolabs). Following electrophoresis in a 1% agarose gel stained with SYBR Safe (Invitrogen), digested DNA was transferred onto Hybond N+ membrane (GE Healthcare, UK). Blots were hybridized with a digoxygenin (DIG)-labelled specific DNA probe for the *HYG* gene and signals were detected using CDP-Star according to the manufacturer’s instructions (Roche Diagnostics).

### Steroid induction of gene expression

Dexamethasone (DEX) was prepared as a 10 mM stock solution in dimethyl sulfoxide (DMSO) and stored at − 20 °C. For each treatment, either DEX (induction) or the equivalent volume of DMSO (mock/control condition) were added to obtain desired concentrations. For application to in vitro cultured seedlings, plants were grown on half concentration Murashige and Skoog medium [32] (½ MS medium) supplemented with 15 g/L sucrose. DEX was added to the medium after autoclaving to obtain final working concentrations of 0.01, 0.1, 1, 5, 10 and 30 μM. Before plating, dehulled seeds were surface sterilized with 70% ethanol for 2 min followed by a 15 min wash with a ~ 2.5% sodium hypochlorite (Fisher Scientific), 0.1% Triton X-100 solution and five washes with sterile water. The plates were incubated with a 16 h/8 h photoperiod, 30 °C day/25 °C night temperatures. Whole seedling induction by submergence was performed in an aqueous solution containing 10 μM DEX and 0.1% Tween-20. Plants grown for 7 days ‘in vitro*’*, as described above, were transferred to 50 ml plastic tubes, covered with the induction/mock solution and incubated under the same growth conditions for 19 h.

For callus treatments, callus was obtained from transgenic lines and maintained according to the rice transformation protocol above. To induce GUS activity, DEX was added to the R1 medium at a final concentration of 10 μM.

Isopropyl β-D-1-thiogalactopyranoside (IPTG) was prepared as aqueous 1 M stock solution and added to ½ MS medium after autoclaving to obtain required concentrations. Seeds germinated for 3 days on ½ MS medium were transferred to plates containing 10 μM DEX supplemented with 10 mM IPTG.

DEX, DMSO, IPTG, sucrose, Triton X-100 and Tween-20 were obtained from Sigma-Aldrich and the Murashige and Skoog medium (including vitamins) was provided by Duchefa.

### Histological assays of GUS activity

GUS histological detection was performed as previously described [33] with minor modifications. Whole seedlings were fixed for 1 h in 90% acetone (Fisher Scientific) at -20 °C, rinsed in 100 mM phosphate buffer, pH 7.6 and stained using 1 mg/ml 5-bromo-4-chloro-3-indolyl-ß-D-glucuronic acid (Melford) supplemented with 0.1% Triton X-100 and 2 mM ferrocyanide/ferricyanide salts. Samples were vacuum infiltrated for 10 min before incubation at 37 °C for 15 h. Callus samples were fixed for 15–20 min and then stained for 4 h using the staining solution described above without the addition of Triton-X-100.

### Enzymatic assays of GUS activity (MUG assay)

GUS (β- glucuronidase) enzymatic activity was measured based on a method described by Jefferson et al. [34]. Extracts were prepared from ground leaf samples using 10 μl protein extraction buffer per mg fresh tissue. Total protein concentration was determined using the Bio-Rad Protein Assay according to the manufacturers protocol for microtiter plates.

The fluorometric reaction was carried out in 96 well-plates (FLUOTRACtm 200) at 37 °C using protein extraction buffer supplemented with 1 mM 4-methylumbelliferyl ß-D-glucuronide (4-MUG) as a substrate. The fluorophore 4-methyl umbelliferone (4-MU) produced upon 4-MUG hydrolysis by ß-glucuronidase (GUS) was quantified by measuring emission at 455 nm using a FLUOstar Omega Microplate Reader set at 365 nm excitation wavelength. A standard curve of 4-MU was used to calculate the amount of 4-MU in each sample. Wild type rice protein was added to the standard curve to correct for eventual autofluorescence or quenching. Activity was calculated from three technical replicates and expressed in (pmoles 4-MU) min^− 1^ (μg protein)^-1.^ 4-MUG and 4-MU stock powders were purchased from Sigma-Aldrich.

### Statistical analysis

Enzymatic activity data and statistical analysis results are provided in Additional file 7. Statistical analysis was performed using RStudio (www.rstudio.com). Due to the nature of the data (unpaired, uneven sample sizes) both a non-parametric one-sided Wilcoxon rank-sum test and a parametric Welch Two Sample t-test were used to test whether the presence of enhancers in the constructs will result in higher GUS activity. Shapiro-Wilk normality tests were used to test for normal distribution and data was log-transformed to normalize the distribution before running the Welch Two Sample t-test. A paired t-test was used to asses differences in GUS activity observed in the *pZmUbi*:*GUS* line with or without DEX treatment. The detailed analysis is included in the supporting information (Additional file 9).

### Bioinformatic screening for the presence of *lacO-*related sequences

The rice reference genome v7.0 downloaded from Phytozome [35, 36] was scanned for sequences matching any of the three *lacI* wild type binding sites, with up to 5 mismatches. The analysis was run using MATLAB release 2018b. A brute force scan was adopted with a sliding window equal in length to the searched sequence that slides one base at every step of scanning. At any base of the reference genome, if the reverse complement of the searched sequence matched the forward strand, the genome sequence going backwards from that base was counted as a match on the reverse strand. To find promoter regions, genes in proximity of the detected matches were identified. If an annotated gene was on the same strand as the match and started within 1000 bases downstream of the match’s first base, then the match was defined as being a putative promoter sequence. MATLAB files and scripts are provided as Additional files 10, 11, 12, 13 and 14.

## Supplementary information

**Additional file 1: Figure S1.** Golden Gate compatible *pOp6*/LhGR level 0 modules **A)** The *pOp6* inducible promoter contains six *lac operator* sequences highlighted in grey. Sequence is flanked by the level 0 PU fusion sites, ***ggag/aatg***. **B)** Rice codon optimized version of the chimeric transcription activator *LhGR* with recognition sites for *BpiI* and *Esp3I* marked in bold italics. Codons are underlined and the domesticated versions, that in each case a contain a silent base pair change, are marked in red. The amino acid encoded by each triplet is specified in brackets. Sequence is flanked by the level 0 SC fusion sites, ***aatg/gctt****.*

**Additional file 2: Data File S1.** Sequence file of construct 17203.

**Additional file 3: Data File S2.** Sequence file of construct 17610.

**Additional file 4: Data File S3.** Sequence file of construct 17613.

**Additional file 5: Figure S2**. DNA gel blot analysis of transgenic lines. **A, B)** Hybridization of *HindIII* digested genomic DNA from T0 transgenic plants (A) and their T1 progeny (B) using a DIG-labelled fragment of the *HYG* gene as a probe. In (A), numbers identify T0 plants resulting from the same transformation event (e.g. 17203_5.1 and 5.2) and in (B) segregating individuals labelled with the same parental line number (e.g. 17203_7.2 A and B) are segregating progeny from that line. The letters in brackets identify corresponding lines shown in Fig. 2. Images originating from the same blot are indicated and numbers distinguish the independent blots used. Arrowheads mark the position of less visible bands. **C**) Position of the *HindIII* restriction site in each construct relative to *HYG*.

**Additional file 6: Table S1.** Summary of T-DNA insertion numbers in each transgenic line tested.

**Additional file 7: Table S2.** GUS enzyme activity in transgenic lines. For each transgenic line, results were obtained from three technical replicates and are expressed as pmoles 4-MU min^− 1^ μg^− 1^. The table also contains a summary of statistical results.

**Additional file 8: Table S3.** List of Golden Gate modules used.

**Additional file 9:** Statistical analysis (RStudio).

**Additional file 10: Data File S4**. MATLAB data file containing *lacO* sequence information. (https://www.mathworks.com/products/matlab.html).

**Additional file 11: Data File S5.** MATLAB data file containing information on rice genes. (https://www.mathworks.com/products/matlab.html).

**Additional file 12: Data File S6.** MATLAB script to search genome for given sequence.

**Additional file 13: Data File S7.** MATLAB script to run the screening experiment on the rice genome.

**Additional file 14: Data File S8.** MATLAB script to generate results table.

## Data Availability

All data generated or analysed during this study are included in this published article [and its additional files].

## Abbreviations

4-MU: 4-methyl umbelliferone
4-MUG: 4-methylumbelliferyl ß-D-glucuronide
DEX: Dexamethasone
GUS: *β-glucuronidase*
HYG: *hygromycin phosphotransferase*
IPTG: β-D-1-thiogalactopyranoside
LhGR: Chimeric transcription factor
pOp6: Inducible promoter based on the *lac* operator (*lacO*) sequence
pOsACT: Rice actin promoter
pZmUbi: Maize ubiquitin promoter
rco: Rice codon-optimized
