# Supplementary material for: A modular steroid-inducible gene expression system for use in rice
Source: BMC Plant Biol. 2019 Oct 15;19:426. doi: 10.1186/s12870-019-2038-x (PMC6794914; doi:10.1186/s12870-019-2038-x)
Supplement: Supplementary file 1 — Additional file 1: Figure S1. Golden Gate compatible pOp6/LhGR level 0 modules A) The pOp6 inducible promoter contains six lac operator sequences highlighted in grey. Sequence is flanked by the level 0 PU fusion sites, ggag/aatg. B) Rice codon optimized version of the chimeric transcription activator LhGR with recognition sites for BpiI and Esp3I marked in bold italics. Codons are underlined and the domesticated versions, that in each case a contain a silent base pair change, are marked in red. The amino acid encoded by each triplet is specified in brackets. Sequence is flanked by the level 0 SC fusion sites, aatg/gctt. [file 12870_2019_2038_MOESM1_ESM.pdf]

A
>pL0M-PU-pOp6
ggagAATTGTGAGCGCTCACAATTGAAAGACTAGAAAGAAGAAAGGGAAGAGAAAGAATTGTGAGCGCTCA
CAATTGAAAGACTAGAAAGAAGAAAGGGAAGAGAAAGAATTGTGAGCGCTCACAATTGAAAGACTAGAAAG
AAGAAAGGGAAGAGAAAGAATTGTGAGCGCTCACAATTGAAAGACTAGAAAGAAGAAAGGGAAGAGAAAGA
ATTGTGAGCGCTCACAATTGAAAGACTAGAAAGAAGAAAGGGAAGAGAAAGAATTGTGAGCGCTCACAATT
GAAAGACTAGTGGATCGATCTTCGCAAGACCCTTCCTCTATATAAGGAAGTTCATTTCATTTGGAGAGGAC
ACG***aatg***

B
>pL0M-SC-rcoLhGR
aatgGCATCAGAGGCACGTAAGACTAAGAAGAAGATCAAAGGAATTCAGCAGGCAACAGCAGGAGTGAGCC
AAGACACCTCTGAGAATCCGAATAAGACGATAGTGCCGGCCGCTCTGCCCCAACTGACTCCGACACTTGTG
TCACTGCTGGAGGTAATAGAGCCGGAGGTCTTGTATGCCGGCTACGATTCGTCCGTCCCTGACAGTGCCTG
GCGCATTATGACCACGCTCAACATGCTCGGTGGGCGGCAAGTCATTGCGGCGGTTAAGTGGGCGAAAGCAA
TTCCTGGTTTCAGGAATCTCCATCTCGATGACCAAATGACCCTCCTCCAATACTCCTGGATGTTCCTGATG
GCGTTTGCCCTCGGTTGGCGCAGTTACCGCCAATCAAGCGGAAACTTGCTGTGCTTTGCACCTGACTTGAT
CATCAATGAACAGAGGATGTCGCTGCCGTGCATGTACGACCAGTGCAAACATATGCTCTTCGTGTCCTCCG
AATTGCAGCGCCTTCAAGTGAGCTACGAAGAATACTTGTGCATGAAAACTCTGCTCTTGTTGAGTTCCGTG
CCTAAGGAGGGACTCAAGAGCCAAGAGCTGTTTGATGAAATCAGGATGACATACATCAAGGAGCTCGGGAA
AGCGATCGTAAAGCGCGAGGGTAACTCATCACAGAACTGGCAACGCTTCTACCAGCTGACTAAGTTGCTCG
ATTCCATGCATGAGGTAGTCGAAAACCTCCTGACCTATTGCTTCCAAACCTTCCTCGATAAGACCATGTCT
ATTGAGTTTCCCGAGATGCTCGCAGAGATAATCACGAACCAGATCCCCAAGTACAGCAACGGTAATATAAA
GAAGCTTCTCTTCCATCAGAAATCTACCTCTAAACCAGTGACCTTGTATGACGTGGCGGAGTACGCCGGAG
TCAGTCATCAAACAGTTTCAAGGGTGGTTAACCAGGCGTCCCACGTTTCGGCAAAGACCAGAGAAAAAGTG
GAGGCAGCTATGGCGGAACTGAATTACATTCCAAACCGCGTGGCACAACAACTGGCGGGAAAGCAAAGCCT
CCTTATTGGGGTGGCAAC***GTCTTC***CCTCGCTCTGCATGCCCCTTCTCAGATTGTTGCTGCCATAAAGAGTC
TCC *(Ser)*
GCGCAGACCAGCTCGGTGCAAGTGTGGTTGTTTCCATGGTCGAAAGGTCGGGTGTGGAGGCGTGTAAAGCG
GCCGTGCACAATTTGCTCGCCCAACGCGTTTCGGGCCTCATAATTAATTACCCTCTGGACGATCAGGATGC
GATTGCCGTAGAAGCAGCTTGTACGAACGTCCCGGCGCTCTTCTTGGACGTCAGCGACCAAACCCCGATTA
ATTCAATTATATTCAGCCATGAGGACGGGACG***CGTCTC***GGCGTGGAGCACCTCGTAGCGCTGGGACATCAA
CGC *(Arg)*
CAAATAGCGCTTCTGGCTGGTCCGCTCTCTTCTGTCTCGGCTCGGCTTAGGCTTGCTGGGTGGCATAAGTA
TTTGACACGTAATCAGATCCAACCAATCGCGGAGAGGGAGGGCGACTGGAGTGCTATGTCTGGATTCCAAC
AGACTATGCAAATGCTCAATGAGGGAATAGTGCCTACCGCCATGCTGGTGGCCAATGATCAAATGGCACTT
GGAGCGATGCGCGCGATTACCGAATCAGGGCTTAGAGTCGGGGCTGACATCTCTGTCGTGGGGTATGATGA
CACTGAGGACTCCTCGTGTTATATCCCACCTCTTACGACAATAAAACAGGACTTCC***GTCTTC***TGGGGCAGA
CGC *(Arg)*
CGTCAGTCGACCGCCTTCTTCAACTGAGCCAGGGTCAGGCGGTAAAGGGCAACCAACTCCTGCCCGTGTCT
CTGGTGAAGCGTAAGACTACAAGTGGCTCAGAATTCGCTAATTTCAATCAGTCCGGGAATATAGCGGACTC
GAGCCTTTCCTTCACTTTTACGAACTCGAGTAACGGCCCTAACCTCATCACCACACAAACAAACAGCCAGG
CCCTCTCGCAGCCGATTGC***GTCTTC***AAACGTCCACGATAATTTCATGAACAATGAAATTACCGCTAGCAAA
TCC *(Ser)*
ATAGATGATGGGAACAATTCAAAACCACTGTCACCAGGGTGGACAGATCAGACCGCTTACAACGCTTTCGG
TATCACGACGGGCATGTTTAATACCACTACCATGGACGACGTCTACAATTACCTGTTTGACGATGAGGACA
CACCACCTAATCCGAAGAAGGAGTGA***gctt***

Figure S1. Golden Gate compatible *pOp6*/LhGR level 0 modules A) The *pOp6* inducible promoter contains six *lac operator* sequences highlighted in grey. Sequence is flanked by the level 0 PU fusion sites, ***ggag/aatg***. **B)** Rice codon optimized version of the chimeric transcription activator *LhGR* with recognition sites for *BpiI* and *Esp3I* marked in bold italics. Codons are underlined and the domesticated versions, that in each case a contain a silent base pair change, are marked in red. The amino acid encoded by each triplet is specified in brackets. Sequence is flanked by the level 0 SC fusion sites, ***aatg/gctt.***
