# Supplementary material for: A modular steroid-inducible gene expression system for use in rice
Source: BMC Plant Biol. 2019 Oct 15;19:426. doi: 10.1186/s12870-019-2038-x (PMC6794914; doi:10.1186/s12870-019-2038-x)
Supplement: Supplementary file 5 — Additional file 5: Figure S2. DNA gel blot analysis of transgenic lines. A, B) Hybridization of HindIII digested genomic DNA from T0 transgenic plants (A) and their T1 progeny (B) using a DIG-labelled fragment of the HYG gene as a probe. In (A), numbers identify T0 plants resulting from the same transformation event (e.g. 17203_5.1 and 5.2) and in (B) segregating individuals labelled with the same parental line number (e.g. 17203_7.2 A and B) are segregating progeny from that line. The letters in brackets identify corresponding lines shown in Fig. 2. Images originating from the same blot are indicated and numbers distinguish the independent blots used. Arrowheads mark the position of less visible bands. C) Position of the HindIII restriction site in each construct relative to HYG. [file 12870_2019_2038_MOESM5_ESM.pdf]

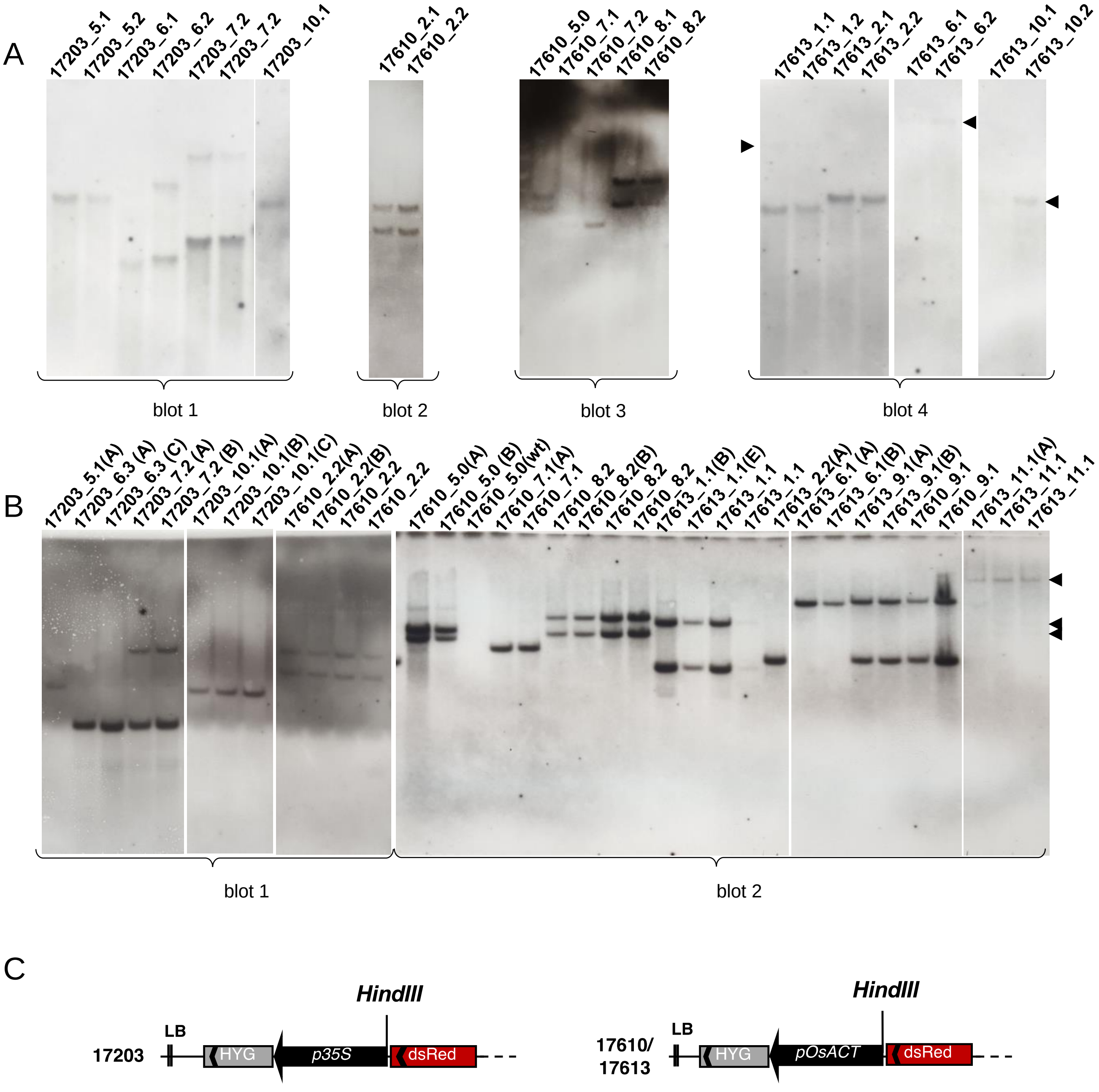

Figure S2. DNA gel blot analysis of transgenic lines. A, B) Hybridization of *HindIII* digested genomic DNA from T0 transgenic plants (A) and their T1 progeny (B) using a DIG-labelled fragment of the *HYG* gene as a probe. In (A), numbers identify T0 plants resulting from the same transformation event (e.g. 17203_5.1 and 5.2) and in (B) segregating individuals labelled with the same parental line number (e.g. 17203_7.2 A and B) are segregating progeny from that line. The letters in brackets identify corresponding lines shown in Figure 2. Images originating from the same blot are indicated and numbers distinguish the independent blots used. Arrowheads mark the position of less visible bands. **C)** Position of the *HindIII* restriction site in each construct relative to *HYG*.
