# Supplementary material for: A modular steroid-inducible gene expression system for use in rice
Source: BMC Plant Biol. 2019 Oct 15;19:426. doi: 10.1186/s12870-019-2038-x (PMC6794914; doi:10.1186/s12870-019-2038-x)
Supplement: Supplementary file 6 — Additional file 6: Table S1. Summary of T-DNA insertion numbers in each transgenic line tested. [file 12870_2019_2038_MOESM6_ESM.pdf]

lines	TDNA insertions in T0	TDNA insertions in T1
17203_5.1	1	1
17203_6.3	1	1
17203_7.2	2	2 (linked)
17203_10.1	1	1
17610_2.2	2	2 (linked)
17610_5.0	2	2 (linked)
17610_7.1	1	1
17610_8.2	2	2 (linked)
17613_1.1	2	2 (linked)
17613_2.2	1	1
17613_6.1	1	1
17613_10.2	1	1
17613_11.1	N/A	3

Table S1. Summary of T-DNA insertion numbers in each transgenic line tested.
