# Supplementary material for: A modular steroid-inducible gene expression system for use in rice
Source: BMC Plant Biol. 2019 Oct 15;19:426. doi: 10.1186/s12870-019-2038-x (PMC6794914; doi:10.1186/s12870-019-2038-x)
Supplement: Supplementary file 7 — Additional file 7: Table S2. GUS enzyme activity in transgenic lines. For each transgenic line, results were obtained from three technical replicates and are expressed as pmoles 4-MU min− 1 μg− 1. The table also contains a summary of statistical results. [file 12870_2019_2038_MOESM7_ESM.pdf]

	Mock					Induced					
**transgenic line**	**technical replicate**	**technical replicate**	**technical replicate**	**mock**	**STDEV.S**	**technical replicate**	**technical replicate**	**technical replicate**	**induced**	**STDEV.S**	**paired t-test p-value**
17203_5A	4.49	5.34	5.90	5.24	0.99	842.08	644.61	1212.59	899.76	261.99	
17203_6A	0.77	0.58	0.77	0.71	0.00	25.06	19.52	31.96	25.51	4.88	
17203_6C	0.42	0.32	0.37	0.37	0.04	53.02	44.34	82.56	59.97	20.89	
17203_6E	0.17	0.24	0.20	0.20	0.02	64.18	63.27	101.00	76.15	26.04	
17203_7A	0.36	0.45	0.34	0.38	0.01	549.07	466.98	997.67	671.24	317.20	
17203_7B	0.41	0.17	0.23	0.27	0.13	30.45	27.33	38.41	32.06	5.63	
17203_7C	4.74	3.77	4.68	4.40	0.04	310.36	298.45	466.60	358.47	110.48	
17203_10A	1.52	1.08	1.74	1.44	0.15	385.77	296.59	579.70	420.69	137.13	
17203_10B	4.89	4.08	5.87	4.95	0.69	642.29	463.99	965.32	690.53	228.42	
17203_10C	2.57	1.95	3.02	2.52	0.32	545.60	418.78	787.76	584.05	171.23	
17610_2A	40.38	24.67	36.75	33.93	2.56	1730.37	1108.59	2725.09	1854.69	703.38	
17610_2B	13.80	13.33	19.11	15.41	3.75	911.31	629.38	1277.02	939.24	258.60	
17610_2D	1.56	1.72	2.15	1.81	0.42	1582.91	1043.92	2024.91	1550.58	312.54	
17610_5A	0.15	0.21	-0.11	0.08	0.18	97.88	77.82	122.39	99.36	17.33	
17610_5B	0.34	0.27	0.26	0.29	0.05	274.38	220.55	343.59	279.51	48.94	
17610_7A	0.35	0.35	0.63	0.44	0.20	224.14	177.16	287.49	229.60	44.80	
17610_7B	0.15	0.24	0.25	0.22	0.07	81.84	70.08	108.61	86.84	18.93	
17610_8B	58.56	39.75	70.54	56.28	8.47	1601.20	1042.09	2577.88	1740.39	690.61	
17610_8D	12.89	11.48	18.98	14.45	4.31	350.55	265.42	481.58	365.85	92.65	
17610_8E	179.64	142.00	221.52	181.06	29.61	1536.39	994.83	2425.15	1652.13	628.45	
17613_1A	17.11	12.81	16.23	15.38	0.62	1389.06	917.34	2258.20	1521.53	614.58	
17613_1B	2.44	1.89	2.49	2.27	0.03	403.71	335.01	672.21	470.31	189.86	
17613_1E	4.17	3.61	6.54	4.77	1.67	529.68	376.47	617.21	507.79	61.89	
17613_2A	2.59	2.19	2.57	2.45	0.02	195.31	144.16	246.57	195.35	36.25	
17613_6A	669.94	511.09	740.05	640.36	49.57	461.26	363.59	631.73	485.53	120.54	
17613_6B	0.79	0.59	1.22	0.87	0.30	135.56	107.06	178.63	140.42	30.45	
17613_9A	0.23	0.16	0.05	0.15	0.13	301.28	229.91	426.47	319.22	88.52	
17613_9C	0.51	0.61	0.41	0.51	0.08	217.26	164.17	328.16	236.53	78.42	
17613_9D	0.46	0.09	0.29	0.28	0.12	76.35	51.06	85.85	71.09	6.72	
17613_9E	0.56	0.34	0.41	0.44	0.11	50.48	33.72	59.89	48.03	6.65	
17613_10A	1.68	1.39	1.66	1.58	0.01	103.84	70.26	125.61	99.90	15.40	
17613_10B	-0.81	0.08	-1.13	-0.62	0.22	32.03	19.67	38.19	29.96	4.36	
17613_11A	0.43	0.71	1.05	0.73	0.44	160.90	103.04	203.46	155.80	30.09	
17613_11B	0.30	0.41	0.23	0.31	0.05	147.66	85.35	170.12	134.38	15.89	
17613_11E	8.90	7.59	9.53	8.68	0.45	312.22	258.77	362.38	311.12	35.47	
Ubi:GUSa	565.52	468.31	792.60	608.81	160.57	262.13	229.59	352.71	281.48	64.05	**0.03128**
Ubi:GUSb	351.81	302.13	431.91	361.95	56.64	195.35	194.39	278.96	222.90	59.12	**0.01249**
Ubi:GUSc	526.01	495.30	749.09	590.13	157.74	292.60	285.33	376.49	318.14	59.32	**0.0331**
WT1	-0.01	-0.17	0.09	-0.03	0.07	-0.07	-0.06	0.11	-0.01	0.12	
WT2	0.14	0.12	-0.14	0.04	0.20	0.07	0.10	0.06	0.08	0.00	
WT3	-0.47	0.11	0.21	-0.05	0.48	0.00	-0.06	-0.26	-0.11	0.19	

**17610**	**17613**
1854.69	1521.53
939.24	470.31
1550.58	507.79
99.36	195.35
279.51	485.53
229.60	140.42
86.84	319.22
1740.39	236.53
365.85	71.09
1652.13	48.03
	99.90
	29.96
	155.80
	134.38
	311.12

Wilcoxon rank sum test

data: EC17610 and EC17613
W = 108 **p-value = 0.03546**

Welch Two Sample t-test

data: log(EC17610) and log(EC17613)
t = 2.1257, df = 17.093
p-value = 0.0484

**17203**	**17610**
899.76	1854.69
25.51	939.24
59.97	1550.58
76.15	99.36
671.24	279.51
32.06	229.60
358.47	86.84
420.69	1740.39
690.53	365.85
584.05	1652.13

Wilcoxon rank sum test

data: EC17203 and EC17613
W = 84 **p-value = 0.3217**

Welch Two Sample t-test

data: log(EC17203) and log(EC17610)
t = -1.6097, df = 17.662
p-value = 0.1252

Table S2. GUS enzyme activity in transgenic lines. For each transgenic line, results were obtained from three technical replicates and are expressed as pmoles 4-MU min^{-1} μg^{-1}. The table also contains a summary of statistical results.
