# Supplementary material for: A modular steroid-inducible gene expression system for use in rice
Source: BMC Plant Biol. 2019 Oct 15;19:426. doi: 10.1186/s12870-019-2038-x (PMC6794914; doi:10.1186/s12870-019-2038-x)
Supplement: Supplementary file 8 — Additional file 8: Table S3. List of Golden Gate modules used. [file 12870_2019_2038_MOESM8_ESM.pdf]

Plasmid ID	Standard name	Other names	Addgene plasmid	Donor	Reference
EC47802	pL1V-R1-47802	pICH47802	#48007	Sylvestre Marillonette	Weber et al., 2011
EC47811	pL1V-R2-47811	pICH47811	#48008	Sylvestre Marillonette	Weber et al., 2012
EC47822	pL1V-R3-47822	pICH47822	#48009	Sylvestre Marillonette	Weber et al., 2013
EC47761	pL1V-F4-47761	pICH47761	#48003	Sylvestre Marillonette	Weber et al., 2014
EC49283	pL1M-ELB-4-49283	pICH49283	#48026	Sylvestre Marillonette	Weber et al., 2015
AGM4723	pL2V-4723	AGM4723	#48015	Sylvestre Marillonette	Weber et al., 2016
pICSL4723				Mark Youles	
EC41421	pL0M-T-Nos-1-41421	pICH41421	#50339	Sylvestre Marillonette& Nicola Patron	Engler et al., 2014
EC75111	pL0M-SC-GUS-1-75111	pICH75111	#50327	Sylvestre Marillonette& Nicola Patron	
EC15030	pL1M-R1-p35S-HYG-tNOS-15030			Ben Miller	p35S in Feike et al., 2019
EC15455	pL0M-PU-pZmUBI-intron1-15455			Ben Miller	Feike et al., 2019
EC15216	pL0M-PU-pOsAct1-intron-15216			Ben Miller	Feike et al., 2019
EC15073	pL0M-SC-dsRed-15073			Ben Miller	
EC15069	pL0M-SC-HYG-15069			Ben Miller	

Table S3. List of Golden Gate modules used.
