# Supplementary material for: A modular steroid-inducible gene expression system for use in rice
Source: BMC Plant Biol. 2019 Oct 15;19:426. doi: 10.1186/s12870-019-2038-x (PMC6794914; doi:10.1186/s12870-019-2038-x)
Supplement: Supplementary file 9 — Additional file 9: Statistical analysis (RStudio). [file 12870_2019_2038_MOESM9_ESM.docx]

**Statistical Analysis (RStudio)**

> shapiro.test(EC17610)

Shapiro-Wilk normality test

data: EC17610

W = 0.82643, p-value = **0.0303 - p < 0.05 reject null hypothesis that data are normally distributed**

> shapiro.test(EC17613)

Shapiro-Wilk normality test

data: EC17613

W = 0.67522, p-value = **0.0001367- p < 0.05 reject null hypothesis that data are normally distributed**

> shapiro.test(EC17203)

Shapiro-Wilk normality test

data: EC17203

W = 0.89209, p-value = **0.179 - p > 0.05 cannot reject null hypothesis that data are normally distributed**

> log(EC17610)

[1] 7.525473 6.845071 7.346383 4.598750 5.633038 5.436339

[7] 4.464067 7.461865 5.902223 7.409821 NA NA

[13] NA NA NA

> log(EC17613)

[1] 7.327472 6.153392 6.230069 5.274793 6.185241 4.944638

[7] 5.765881 5.466075 4.263947 3.871826 4.604170 3.399863

[13] 5.048573 4.900668 5.740181

> log(EC17203)

[1] 6.802128 3.239071 4.093844 4.332705 6.509127 3.467609

[7] 5.881845 6.041896 6.537459 6.369987

> shapiro.test(log(EC17610))

Shapiro-Wilk normality test

data: log(EC17610)

W = 0.8683, p-value = **0.09551**

> shapiro.test(log(EC17613))

Shapiro-Wilk normality test

data: log(EC17613)

W = 0.9859, p-value = **0.9948**

> shapiro.test(log(EC17203))

Shapiro-Wilk normality test

data: log(EC17203)

W = 0.84859, p-value = **0.05588**

> t.test(log(EC17610), log(EC17613))

**Welch Two Sample t-test**

data: log(EC17610) and log(EC17613)

t = 2.1257, df = 17.093, p-value = **0.0484**

alternative hypothesis: true difference in means is not equal to 0

95 percent confidence interval:

0.007764608 1.959936243

sample estimates:

mean of x mean of y

6.262303 5.278452

> t.test(log(EC17203), log(EC17610))

**Welch Two Sample t-test**

data: log(EC17203) and log(EC17610)

t = -1.6097, df = 17.662, p-value = **0.1252**

alternative hypothesis: true difference in means is not equal to 0

95 percent confidence interval:

-2.1563641 0.2868925

sample estimates:

mean of x mean of y

5.327567 6.262303

> wilcox.test(EC17610, EC17613,

+ alternative = c( "greater"),

+ paired = FALSE, conf.level = 0.95)

**Wilcoxon rank sum test**

data: EC17610 and EC17613

W = 108, **p-value = 0.03546**

alternative hypothesis: true location shift is greater than 0

> wilcox.test(EC17203, EC17613,

+ alternative = c( "greater"),

+ paired = FALSE, conf.level = 0.95)

**Wilcoxon rank sum test**

data: EC17203 and EC17613

W = 84, p-value = **0.3217**

alternative hypothesis: true location shift is greater than 0

#UbiGUSa mock

> x<-c(565.52, 468.31, 792.6)

> #UbiGUSa DEX induced

> y<-c(262.13, 229.59, 352.71)

> res<-t.test(x, y, paired=TRUE)

> res

**Paired t-test**

data: x and y

t = 5.5205, df = 2, **p-value = 0.03128**

alternative hypothesis: true difference in means is not equal to 0

95 percent confidence interval:

72.21221 582.45445

sample estimates:

mean of the differences

327.3333

> #UbiGUSb mock

> z<-c(351.81, 302.13, 431.91)

> #UbiGUSb DEX

> t<-c(195.35, 194.39, 278.96)

> res<-t.test(z, t, paired=TRUE)

> res

**Paired t-test**

data: z and t

t = 8.8636, df = 2, **p-value = 0.01249**

alternative hypothesis: true difference in means is not equal to 0

95 percent confidence interval:

71.55103 206.54897

sample estimates:

mean of the differences

139.05

> #UbiGUSc mock

> x<-c(526.01, 495.30, 749.09)

> #UbiGUSc DEX

> y<-c(292.6, 285.33, 376.49)

> res<-t.test(x, y, paired=TRUE)

> res

**Paired t-test**

data: x and y

t = 5.3588, df = 2, **p-value = 0.0331**

alternative hypothesis: true difference in means is not equal to 0

95 percent confidence interval:

53.6062 490.3805

sample estimates:

mean of the differences

271.9933
